# Medical expenses and influencing factors in lung cancer patients: a comparative analysis between surgical and non-surgical cases from Xiamen, China

**DOI:** 10.3389/fpubh.2025.1714688

**Published:** 2026-01-19

**Authors:** Linyan Chen, Wenting Luo, Juntong Liu, Minqiang Lin, Zhicheng Zhuang

**Affiliations:** 1 Medical Insurance and Pricing Regulation Department, The First Affiliated Hospital of Xiamen University, School of Medicine, Xiamen University, Xiamen, Fujian, China; 2 Research Department, The First Affiliated Hospital of Xiamen University, School of Medicine, Xiamen University, Xiamen, Fujian, China

**Keywords:** lung cancer, medical expenses, influencing factors, diagnosis-related group, disease-based insurance payment

## Abstract

**Objective:**

To investigate medical expenses and factors influencing surgical choices among lung cancer patients in a tertiary hospital in Xiamen, China, and to identify key cost differences between surgical and non-surgical approaches.

**Methods:**

In this retrospective cross-sectional study, we analyzed 3,806 lung cancer patients treated in 2023. Data analysis was performed using SPSS 27.0, with independent-sample *t*-tests for cost comparisons and binary logistic regression to identify factors influencing surgical intervention.

**Results:**

The study analyzed 3,806 lung cancer patients (60.7% male, 51.1% aged 61–80 years), revealing significant demographic and clinical predictors of surgical intervention. Multivariate analysis identified female gender (adjusted OR = 1.989, 95%CI:1.624–2.436, *p* < 0.001), younger age (61–80 years: adjusted OR = 0.454, 95% CI:0.305–0.676, *p* = 0.001; >80 years: adjusted OR = 0.353, 95%CI: 0.161–0.774, *p* = 0.009), and prolonged hospitalization (adjusted OR = 106.729, 95%CI: 79.485–143.312, *p* < 0.001) as key determinants, while insurance type showed no association. Surgical patients incurred 8.5-fold higher median costs (¥48,610 vs. ¥5,676), with medical consumables exhibiting the most pronounced disparity (>2,000-fold difference). The predictive model demonstrated excellent discrimination (AUC = 0.904), calibration (Hosmer-Lemeshow *p* = 0.402), and specificity (81.6%) at a 46.75% probability threshold, with length of stay being the strongest individual predictor (univariate AIC = 2,874 vs. full model AIC = 2,802). Insurance type showed no significant association with surgical treatment in either univariate or multivariate analyses.

**Conclusion:**

Gender, age, and hospital stay length were major factors associated with surgical decisions in lung cancer patients, with surgery significantly increasing total medical costs. Policy improvements in the management of high-value medical consumables and insurance reimbursement are needed to reduce financial burdens and enhance surgical accessibility.

## Introduction

Lung cancer persists as one of the most prevalent malignancies globally, characterized by high incidence and mortality, with profound impacts on patients’ quality of life, workforce productivity losses, and economic stability. According to the latest Global Cancer Observatory (GLOBOCAN 2022) data, lung cancer accounted for 12.4% of all new cancer cases and 18.7% of cancer-related deaths worldwide in 2022 (1). In China, it remains the leading cause of cancer mortality, with approximately 870,982 new cases and 766,352 deaths in 2022 (2). The economic impact of lung cancer is substantial, affecting healthcare systems, patients, and families alike. Studies have shown that lung cancer leads to significant productivity loss-primarily due to premature mortality and long-term disability-and imposes high out-of-pocket expenses for patients during treatment and follow-up. A comprehensive analysis by Mariotto et al. estimated the annual national expenditure for lung cancer care in the United States exceed $15 billion (3). Similarly, studies from Europe and China have documented average direct medical costs per patient associated with lung cancer treatment; in China specifically, the average direct medical cost per patient can surpass ¥100,000, depending on cancer stage and treatment modality (4, 5).

Treatment strategies for lung cancer include surgery, radiotherapy, chemotherapy, targeted therapy, immunotherapy, and various combinations thereof. The choice of treatment depends on multiple factors, including tumor stage, histological type, patient comorbidities, age, and performance status (6). Surgical intervention, particularly for early-stage disease, remains a cornerstone of curative treatment but is associated with substantial costs. With the implementation of diagnosis-related group (DRG) and disease-based insurance payment (DIP) systems in China, understanding the cost structure and influencing factors of lung cancer treatment has become increasingly important for healthcare resource allocation, policy development, and improving accessibility to care (7). However, comprehensive analyses of the factors influencing treatment choices and their associated costs in the Chinese healthcare context remain limited.

Therefore, this study aims to investigate the medical expenses and influencing factors among lung cancer patients in a tertiary hospital in Xiamen, China, with a particular focus on differences between surgical and non-surgical cases. By identifying key determinants of treatment selection and cost variations, this research seeks to provide evidence-based insights for policymakers to improve healthcare financing efficiency and reduce the economic burden of lung cancer.

## Materials and methods

### Study design and population

This retrospective cross-sectional study analyzed data from lung cancer patients treated at a tertiary hospital in Xiamen, China. We analyzed data from all lung cancer patients treated at the hospital between January 1 and December 31, 2023. Patients were eligible if they had a primary diagnosis of lung cancer (ICD-10 codes C33–C34) and received inpatient treatment at the study site during the specified period. The sample size (*n* = 3,806) represents the complete set of eligible cases hospitalized during the year, thus eliminating the need for additional sample size calculation. The 1-year time frame was chosen to encompass seasonal and case-mix variations, and the number of cases was considered sufficient to reflect the hospital’s lung cancer patient population. Cases with incomplete clinical or cost data were excluded from further analysis.

### Data collection

Clinical and demographic data were extracted from the hospital’s electronic medical record (EMR) system, including gender, age, length of hospital stay, insurance type, and treatment modality. Patients were categorized into surgical and non-surgical groups based on whether they underwent surgical intervention for lung cancer. Cost data were obtained from the hospital’s financial management system, which provides itemized billing records. Expenses were classified according to the hospital’s standard accounting categories: total costs, medical supplies, medications (including Western medicines, Chinese patent medicines, and Chinese herbal medicines), examination and laboratory fees, diagnostic fees, bed fees, nursing fees, treatment fees, and other expenses.

Exclusion criteria: (1) Patients with unclear clinical diagnosis; (2) Incomplete recording of key data, including lack of pathological confirmation, detailed medical expense breakdowns, or core data such as length of hospital stay; (3) Obvious errors in case records, such as discrepancies where the sum of individual expense items did not equal total expenses.

### Statistical analysis

Descriptive statistics were used to summarize patient characteristics and cost data. Continuous variables were expressed as mean ± standard deviation, and categorical variables as frequencies and percentages. Independent samples *t*-tests were employed to compare cost components between surgical and non-surgical groups.

Binary logistic regression analysis was conducted to identify factors influencing the likelihood of receiving surgical treatment. The dependent variable was treatment type (surgical vs. non-surgical), while independent variables included gender, age (categorized as 0–40, 41–60, 61–80, and >80 years), length of hospital stay (categorized as <5, 6–10, 11–15, and >16 days), and insurance type (local insurance, out-of-province insurance within the province, out-of-province insurance from other provinces, and self-pay).

All statistical analyses were performed using SPSS version 27.0. *p*-values <0.05 were considered statistically significant. Odds ratios (ORs) with 95% confidence intervals (CIs) were calculated to quantify the associations between predictor variables and surgical intervention.

## Results

### Characteristics of the study population

After excluding irrelevant cases and missing values, a total of 3,806 samples were included in the study. There were 2,310 male patients (60.7%) and 1,496 female patients (39.3%). Patients aged 61–80 years constituted the largest group with 1,943 cases (51.1%), followed by those aged 41–60 years with 1,534 cases (40.3%).

The lung cancer patients in this study were categorized into four insurance types: local medical insurance, inter-regional medical insurance (within province), inter-regional medical insurance (out of province), and self-pay. Patients with local medical insurance numbered 1,823 cases, accounting for 47.9% of all lung cancer patients. Inter-regional medical insurance patients (within province) ranked second with 1,693 cases, representing 44.5% of all lung cancer patients. Self-pay patients were the least numerous, with only 51 cases, accounting for 1.3%.

The majority of patients did not receive surgery (2,214 patients, 58.0%), while 1,952 patients (42.0%) underwent surgical treatment. Regarding length of hospital stay, short-term hospitalization was the most common category, with 1,949 patients (51.2%), followed by >16 days, accounting for 16.7%. The comparative analysis of cost variables between non-surgical and surgical patients revealed statistically significant differences (all *p* < 0.001) across all financial metrics. Surgical patients demonstrated substantially higher median costs, with total hospitalization expenses being approximately 8.5 times greater (¥48,609.71 vs. ¥5,676.27) than non-surgical cases. This cost disparity was particularly pronounced in medical consumables (¥19,570.87 vs. ¥8.94, >2,000-fold difference) and examination/laboratory fees (¥7,156.50 vs. ¥1,673.50). Pharmaceutical costs were also significantly elevated for surgical patients (¥4,521.62 vs. ¥2,770.06), though with a smaller magnitude of difference. The actual settlement amount followed a similar pattern, with surgical patients paying nearly 8.5 times more (¥48,250.00 vs. ¥5,662.19). These findings collectively demonstrate that surgical intervention is associated with dramatically increased healthcare expenditures across all measured cost categories, with medical consumables representing the most disproportionately affected expense sector. Details are shown in Table 1.

**Table 1:** Characteristics of the study population.

Variables	Non-surgical patients (*n* = 2,214)	Surgical patients (*n* = 1,592)	*P*-value*
Age	63.00 [56.00, 69.00]	60.00 [52.00, 68.00]	<0.001
Length of stay, days	1.03 [0.27, 3.12]	13.01 [8.91, 18.98]	<0.001
By item total cost, ¥	5676.27 [3112.98, 8485.80]	48609.71 [21989.69, 63968.00]	<0.001
Pharmaceutical fee, ¥	2770.06 [500.00, 5184.60]	4521.62 [2950.45, 6666.17]	<0.001
Medical consumables fee, ¥	8.94 [4.17, 23.18]	19570.87 [1488.46, 30467.02]	<0.001
Examination and laboratory fee, ¥	1673.50 [936.00, 2789.25]	7156.50 [5109.75, 10603.50]	<0.001
Actual settlement amount, ¥	5662.19 [3126.35, 8481.15]	48250.00 [2,530.15, 62919.56]	<0.001
Gender			<0.001
Female	692 (31.3%)	804 (50.5%)	
Male	1,522 (68.7%)	788 (49.5%)	
Age group			<0.001
<40 years	118 (5.3%)	147 (9.2%)	
41–60 years	848 (38.3%)	686 (43.1%)	
61–80 years	1,213 (54.8%)	730 (45.9%)	
>80 years	35 (1.6%)	29 (1.8%)	
Length of stay group			<0.001
<5 Days	1806 (81.6%)	143 (9.0%)	
6–10 Days	214 (9.7%)	388 (24.4%)	
11–15 Days	113 (5.1%)	432 (27.1%)	
>16 Days	81 (3.7%)	629 (39.5%)	
Insurance type group			0.79
Inter-regional (out of province)	135 (6.1%)	104 (6.5%)	
Inter-regional (within province)	994 (44.9%)	699 (43.9%)	
Local medical insurance	1,053 (47.6%)	770 (48.4%)	
Self-pay	32 (1.4%)	19 (1.2%)	

* *P*-values were calculated using Mann–Whitney *U* test for continuous variables and Chi-square test (or Fisher’s exact test) for categorical variables.

### Surgical status of lung cancer patients

This study included a total of 3,806 samples, comprising 1,592 surgical patients and 2,214 non-surgical patients. Among all surgical patients, female patients (804 cases) slightly outnumbered male patients; whereas among non-surgical patients, males (1,522 cases) far exceeded females (692 cases). Chi-square test results indicated a significant difference between gender and surgical choice (*p* < 0.001), suggesting that gender significantly influences surgical decisions.

**Figure 1:**
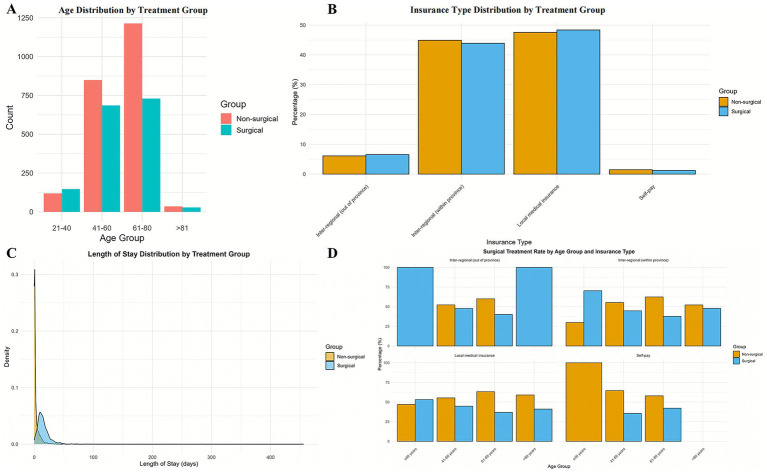
Distribution patterns of age, length of stay, and insurance type with intergroup comparison. Data are presented as *n* (%): non-surgical group (*n* = 2,214) versus surgical group (*n* = 1,592). *p*-values from Pearson tests. **(A)** Age group distribution: Significant disparity across cohorts (*p* < 0.001), with 61–80 years being the predominant group (54.8% vs. 45.9%). **(B)** Length of stay (LOS) stratification: Highly divergent patterns (*p* < 0.001); 81.6% of Group 1 had <5 days LOS vs. 39.5% of Group 2 with >16 days. **(C)** Insurance type composition: No significant intergroup difference (*p* = 0.79), with local medical insurance being most common (47.6% vs. 48.4%). **(D)** This figure displays the age-stratified distribution of insurance types.

In terms of age distribution, the 61–80 age group had the largest number of lung cancer patients. Within this age group, 45.9% of patients opted for surgery. The 41–60 age group ranked second in the number of surgical lung cancer patients but had the highest proportion of surgical patients at 43.1%. Chi-square test results showed significant differences in surgery rates among lung cancer patients of different age groups (*p* < 0.001), indicating that age significantly impacts surgical choice (Figure 1).

The difference in numbers of surgical and non-surgical patients varied greatly according to length of hospital stay. Chi-square test results showed a significant difference between length of stay and surgical choice (*p* < 0.001). Non-surgical patients were concentrated in short-term hospitalizations, with 1,806 patients staying <5 days and only 143 surgical patients in this category; whereas surgical patients increased with extended hospitalization times, with surgical patients far outnumbering non-surgical patients when hospital stays exceeded 16 days. This indicates that longer hospital stays are associated with higher likelihood of surgical intervention.

Based on chi-square test results, significant differences in surgery rates exist among lung cancer patients of different genders, ages, and lengths of hospital stay (*p* < 0.05), with higher surgery rates observed among females, younger to middle-aged patients, and those with longer hospital stays. However, differences in surgery rates among patients with different insurance types were not statistically significant (*p* = 0.79), indicating that insurance type does not significantly affect surgical choice (Table 1). Details of normal distribution test results are shown in Table 2.

**Table 2:** Normal distribution test results.

Variables	Group	Mean (Standard deviation)	Shapiro–Wilk	*P*-value	Normality
Age, years	Non-surgical	61.63 ± 10.32	0.981	<0.001	No
	Surgical	58.96 ± 12.16	0.982	<0.001	No
Length of stay, days	Non-surgical	3.23 ± 6.32	0.485	<0.001	No
	Surgical	15.91 ± 18.13	0.410	<0.001	No
Total cost, ¥	Non-surgical	7517.45 ± 9333.44	0.556	<0.001	No
	Surgical	46879.88 ± 32338.58	0.737	<0.001	No
Pharmaceutical fee, ¥	Non-surgical	3861.52 ± 4781.12	0.718	<0.001	No
	Surgical	6126.23 ± 10826.79	0.306	<0.001	No
Medical consumables fee, ¥	Non-surgical	64.10 ± 270.71	0.213	<0.001	No
	Surgical	18446.43 ± 15469.21	0.907	<0.001	No
Examination and laboratory fee, ¥	Non-surgical	2145.48 ± 2121.50	0.762	<0.001	No
	Surgical	8540.09 ± 6128.52	0.662	<0.001	No
Actual settlement amount, ¥	Non-surgical	7524.98 ± 9348.63	0.560	<0.001	No
	Surgical	47022.80 ± 32436.52	0.738	<0.001	No

All variables showed non-normal distribution by Shapiro–Wilk test (*p* < 0.001).

**Figure 2:**
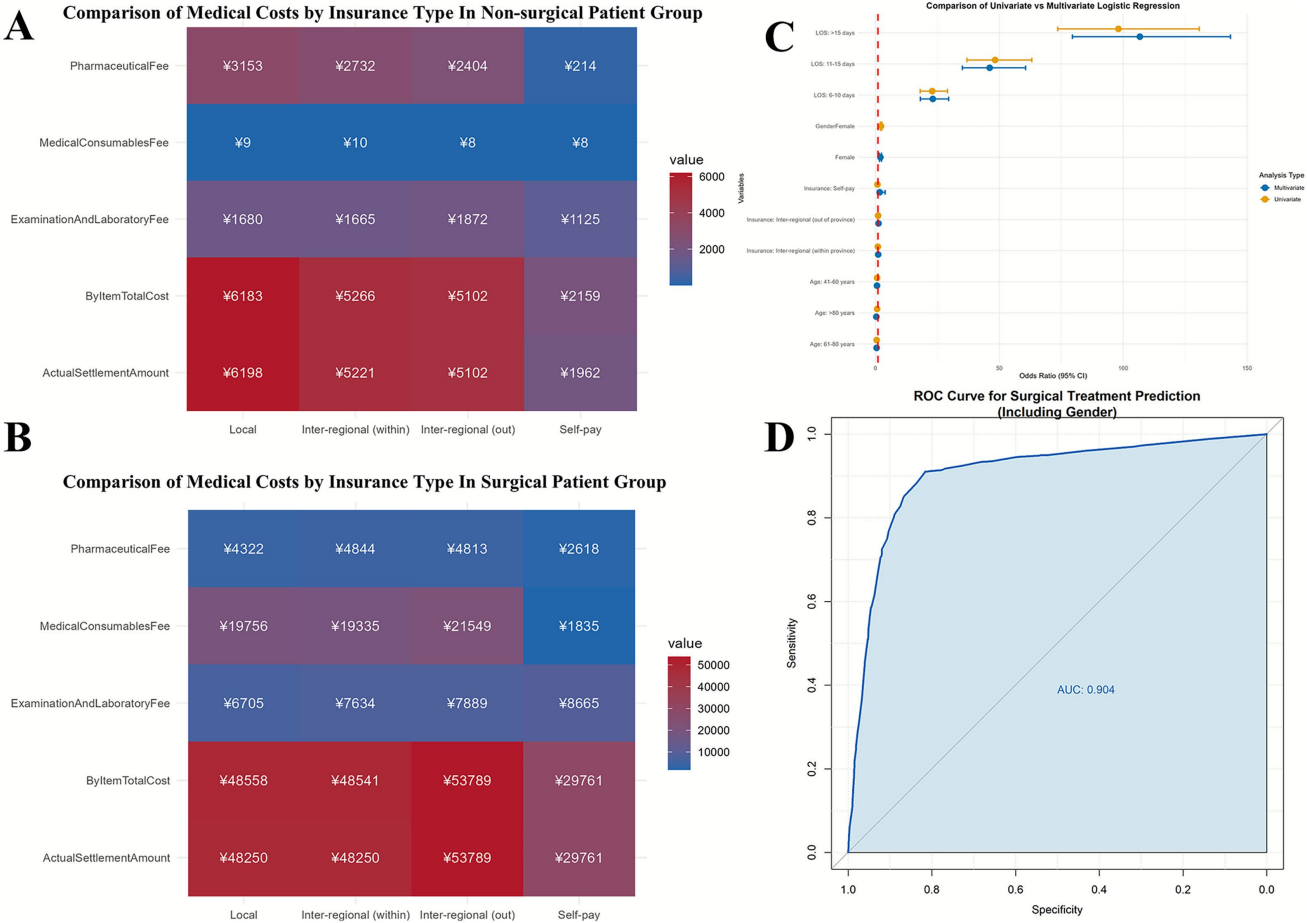
Comparative analysis of medical costs by insurance type and predictive performance of multivariate logistic regression model. **(A,B)** Medical cost comparison by insurance type between surgical and non-surgical patient groups. **(C)** Forest plot displaying odds ratios (ORs) with 95% confidence intervals for both univariate and multivariate analyses. Key covariates: Length of stay (LOS). Reference lines indicate null effect (OR = 1). **(D)** Receiver operating characteristic (ROC) curve of the final model (solid blue line; AUC = 0.904 [95% CI: 0.894–0.915]), compared to baseline (dashed gray line). Optimal cutoff: sensitivity 91.02%, specificity 81.62%.

The Kruskal-Wallis test demonstrated significant cost disparities across insurance types for non-surgical patients (all *p* < 0.05), with local medical insurance associated with the highest total costs (¥6183.27 [3603.08–9504.43]) and self-pay the lowest (¥2159.46 [651.18–4408.00]). Surgical patients showed less variation, though consumables fees differed significantly (*p* = 0.013), with inter-regional (out-of-province) patients incurring the highest costs (¥21548.75 [2137.35–32016.54]). Notably, pharmaceutical expenses varied most dramatically among non-surgical self-pay patients (¥214.26 [36.20–2915.65] vs. ¥3152.51 [687.00–6150.29] for local insurance). These findings highlight insurance-type-driven economic burdens in non-surgical care, while surgical costs appear more homogenized, possibly due to standardized procedural pricing (Table 3 and Figures 2A,B).

**Table 3:** Comparison of medical costs by insurance type and patient group (surgical vs. non-surgical).

Group	Cost, ¥	Insurance type					
		Inter-regional (out of province)	Inter-regional (within province)	Local medical insurance	Self-pay	χ^2^	*P*-Value*
Non-surgical patients (*n* = 2,214)	By item total cost, ¥	5102.26 (3208.81–7733.59)	5265.61 (2847.49–7873.74)	6183.27 (3603.08–9504.43)	2159.46 (651.18–4408.00)	50.58	<0.001
	Pharmaceutical fee, ¥	2403.80 (376.74–5297.84)	2732.17 (398.49–4430.33)	3152.51 (687.00–6150.29)	214.26 (36.20–2915.65)	45.57	<0.001
	Medical consumables fee, ¥	7.54 (2.97–21.43)	10.05 (5.42–25.38)	8.59 (3.08–21.31)	8.32 (6.01–24.35)	14.09	0.00278
	Examination and laboratory fee, ¥	1872.00 (831.00–2854.50)	1665.00 (929.25–2812.75)	1680.00 (1013.00–2791.00)	1125.00 (0.00–1986.25)	7.84	0.0495
	Actual settlement amount, ¥	5102.26 (3208.81–7733.59)	5221.41 (2838.49–7842.38)	6197.65 (3557.17–9525.70)	1961.62 (347.81–4408.00)	52.9	<0.001
							
Surgical patients (*n* = 1,592)	By item total cost, ¥	53788.98 (21222.73–64882.16)	48540.54 (18852.82–65212.60)	48557.60 (29297.74–61872.62)	29760.55 (12788.04–50907.94)	4.38	0.2236
	Pharmaceutical fee, ¥	4812.88 (2947.11–6463.40)	4844.48 (2911.16–7055.30)	4321.62 (3019.94–6186.07)	2617.98 (1061.66–4935.21)	6.28	0.0987
	Medical consumables fee, ¥	21548.75 (2137.35–32016.54)	19335.20 (1300.10–31681.54)	19756.31 (2386.65–29609.61)	1835.28 (565.56–11190.14)	10.73	0.0133
	Examination and laboratory fee, ¥	7889.00 (5544.75–10858.50)	7634.00 (5311.50–10822.00)	6705.00 (4929.00–10117.50)	8665.00 (5203.00–16275.00)	7.17	0.0666
	Actual settlement amount, ¥	53788.98 (20946.33–64882.16)	48250.00 (18979.36–64443.59)	48250.00 (29371.25–61550.00)	29760.55 (12788.04–50907.94)	5.32	0.1496

*The Kruskal-Wallis test revealed statistically significant differences among groups.

### Factors influencing surgical intervention

Table 4 presents the results of the univariate and multivariate logistic regression analysis examining factors associated with receiving surgical treatment. Female patients exhibited nearly twice the odds of undergoing surgery compared to males (adjusted OR = 1.989, 95%CI:1.624–2.436, *p* < 0.001). A strong inverse relationship was observed with age, where patients aged 61–80 years showed 55% lower odds (adjusted OR = 0.454, 95% CI:0.305–0.676, *p =* 0.001) and those over 80 years had 65% lower odds (adjusted OR = 0.353, 95%CI: 0.161–0.774, *p =* 0.009) compared to the youngest reference group (0–40 years). Most strikingly, length of hospital stay demonstrated a dose-dependent effect, with patients hospitalized for >16 days exhibiting 107-fold higher odds of surgical intervention (adjusted OR = 106.729, 95%CI: 79.485–143.312, *p* < 0.001) relative to those with stays <5 days. Insurance type showed no significant association with surgical treatment in either univariate or multivariate analyses. These results highlight length of hospitalization as the strongest predictor, followed by gender and age, in determining surgical treatment decisions.

**Table 4:** Univariate and multivariate logistic regression analysis of factors associated with surgical treatment.

Variables	Univariate analysis					Multivariate analysis	
	Coefficient	SE	*Z*-value	OR (95%CI)	*P*-value	OR (95%CI)	*P*-value
Gender (Male as reference)							
Female	0.81	0.068	11.90	2.244 (1.964–2.564)	<0.001	1.989 (1.624–2.436)	<0.001
Age (0–40 years as reference)							
41–60 years	−0.43	0.134	−3.23	0.649 (0.500–0.844)	0.001	0.616 (0.415–0.914)	0.016
61–80 years	−0.73	0.132	−5.50	0.483 (0.373–0.626)	<0.001	0.454 (0.305–0.676)	0.001
>80 years	−0.41	0.280	−1.46	0.665 (0.384–1.151)	0.15	0.353 (0.161–0.774)	0.009
Length of hospital stay (<5 days as reference)							
6–10 Days	3.13	0.122	25.74	22.898 (18.041–29.063)	<0.001	23.145 (18.133–29.544)	0.001
11–15 Days	3.88	0.137	28.34	48.282 (36.928–63.128)	<0.001	46.108 (35.073–60.615)	0.001
>16 Days	4.59	0.147	31.29	98.073 (73.585–130.709)	<0.001	106.729 (79.485–143.312)	0.001
Insurance type (Local medical insurance as reference)							
Inter-regional insurance (within province)	0.052	0.139	0.38	1.054 (0.803–1.383)	0.71	-	-
Inter-regional insurance (out of province)	−0.04	0.068	−0.57	0.962 (0.841–1.100)	0.57	-	-
Self-pay	−0.21	0.293	−0.71	0.812 (0.457–1.443)	0.48	-	-

SE: Standard Error.

The logistic regression model demonstrated excellent predictive performance with an AUC of 0.904 (95% CI: 0.894–0.915), indicating strong discriminative ability between surgical and non-surgical cases. Model fit was confirmed by a significant likelihood ratio test (χ^2^ = 582.45, *p* < 0.001) and good calibration (Hosmer-Lemeshow test: χ^2^ = 8.32, *p* = 0.402). At the optimal cutoff of 46.75%, the model achieved high sensitivity (91.02%) and specificity (81.62%), yielding a Youden Index of 72.63%. The model showed moderate-to-good explanatory power (Nagelkerke *R*^2^ = 0.63) with no multicollinearity concerns (all VIFs<1.15). The multivariate model (AIC = 2802.13) significantly outperformed all univariate models, with length of hospital stay emerging as the strongest individual predictor (univariate AIC = 2874.28). These results validate the model’s robustness for surgical treatment prediction while highlighting key clinical predictors (Table 5 and Figures 2C,D).

**Table 5:** Logistic regression model diagnostics, fit statistics and model comparison.

Diagnostic index	Value		Interpretation/Threshold	
Model fit				
Likelihood Ratio Test	χ^2^ = 582.45		*P* < 0.001	
Hosmer-Lemeshow Test	χ^2^ = 8.32		*P* = 0.402, Good fit	
ROC AUC	0.9040 (95% CI: 0.8936–0.9145)			
Sensitivity	91.02%		True positive rate at optimal cutoff	
Specificity	81.62%		True negative rate at optimal cutoff	
Optimal cutoff	46.75%		Predicted probability threshold for surgical treatment	
Youden Index	72.63%		Sensitivity + Specificity - 1 (higher is better)	
Explanatory power (Pseudo *R*^2^)				
McFadden *R*^2^	0.46		Moderate fit	
Cox-Snell *R*^2^	0.47		Weak to moderate fit	
Nagelkerke *R*^2^	0.63		Moderate to good fit	
Multicollinearity				
Age	VIF = 1.147		No issue (VIF < 5)	
Length of hospital stay	VIF = 1.046		No issue (VIF < 5)	
Insurance type	VIF = 1.074		No issue (VIF < 5)	
Gender	VIF = 1.079		No issue (VIF < 5)	
Model comparison				
Univariate (Age)	AIC = 5142.02	BIC = 5167.00	LogLik = −2567.01	Delta_AIC = 2339.89
Univariate length of hospital stay	AIC = 2874.28	BIC = 2899.25	LogLik = −1433.14	Delta_AIC = 72.15
Univariate (Insurance Type)	AIC = 5181.08	BIC = 5206.05	LogLik = −2586.54	Delta_AIC = 2378.95
Univariate (Gender)	AIC = 5034.52	BIC = 5047.01	LogLik = −2515.26	Delta_AIC = 2232.40
Multivariate (Full)	AIC = 2802.13	BIC = 2870.81	LogLik = −1390.06	Delta_AIC = 0.00

AIC: Akaike Information Criterion; AUC: Area Under the ROC Curve; BIC: Bayesian Information Criterion; CI: Confidence Interval; LogLik: Log-Likelihood; R^2^: Pseudo R-squared; ROC: Receiver Operating Characteristic; VIF: Variance Inflation Factor.

## Discussion

This study provides a comprehensive analysis of medical expenses and influencing factors among lung cancer patients, with a particular focus on differences between surgical and non-surgical cases. Our findings have important implications for healthcare resource allocation, policy development, and clinical practice.

Our results demonstrated that medical supply expenses were the primary driver of cost differences between surgical and non-surgical patients, with surgical patients incurring costs higher than non-surgical patients. This finding aligns with previous studies in various healthcare settings. Tsevat et al. (8) analyzed cost components in thoracic surgery and identified disposable instruments, stapling devices, and energy devices as major contributors to high surgical costs. Similarly, Swanson et al. (9) reported that consumable surgical supplies accounted for approximately 30–40% of total costs in video-assisted thoracoscopic surgery (VATS) for lung cancer. The high cost of surgical supplies can be attributed to several factors. First, minimally invasive thoracoscopic procedures, which have become standard practice for lung cancer surgery, require specialized instruments and devices that are often expensive (10). Second, the complexity of lung resection necessitates multiple staplers, energy devices, and hemostatic agents (11). Third, many high-value consumables are produced by a limited number of manufacturers, leading to less competitive pricing (12).

Our study also revealed the underscore a critical divergence in economic burden: non-surgical care exhibits significant insurance-type-dependent cost variation, whereas surgical expenditures demonstrate relative homogeneity, likely attributable to institutionalized procedural pricing frameworks. The observed cost disparities between insurance types reveal fundamental structural inequities within China’s universal coverage system. While our data confirm that 95% of the population is nominally insured through three primary schemes-Urban Employee Basic Medical Insurance (UEBMI), Urban Resident Basic Medical Insurance (URBMI), and New Rural Cooperative Medical Scheme (NRCMS) (13)-the reimbursement depth varies drastically. Surgical costs exhibited relative homogeneity across insurance types (e.g., only 1.2-fold difference between UEBMI and NRCMS recipients), likely reflecting standardized procedural pricing under Diagnosis-Related Group (DRG) reforms (14). In stark contrast, non-surgical care costs varied up to 3.5-fold, with local insurance patients incurring median costs of ¥6,183 (IQR ¥3,603–9,504) versus ¥2,159 (IQR ¥651–4,408) for self-pay patients. This divergence aligns with Liu et al. (15), findings that non-surgical oncology treatments lack effective cost-control mechanisms under current policies. The financial burden disparity is most acute for catastrophic illnesses: a lung cancer patient under NRCMS would pay 48–56% of total costs out-of-pocket (median ¥280,000) compared to 10–16% for UEBMI enrollees (¥50,000–80,000) (16). This 5-fold difference exacerbates existing urban–rural health inequities, as 72% of NRCMS beneficiaries reside in rural areas with lower incomes (17). Systemic causes include fragmented financing pools (UEBMI’s per-capita funding is 6 × higher than NRCMS (18)) and restricted cross-scheme portability, disproportionately affecting China’s 290 million migrant workers (19). These findings underscore the urgent need for benefit package harmonization, particularly for chronic and neoplastic diseases where non-surgical modalities dominate long-term management.

China’s transition from fee-for-service to DRG/DIP-based payment systems, accelerated since the 2019 national mandate, has profoundly reshaped hospital incentive structures and cost containment strategies. Our finding that surgical consumables dominate cost differentials (constituting 40.3% of total surgical expenses vs. 0.16% for non-surgical cases) directly reflects the challenges of controlling high-value device expenditures under current DRG groupings. Wang et al. (20) analyzed DRG payment data from 128 hospitals and identified “surgical high-value consumables” as the primary driver of DRG weight outliers, with lung resections exceeding payment caps by 18–35% in 42% of cases. This creates perverse incentives for hospitals to either (1) select lower-risk patients who require fewer expensive devices, potentially limiting access for complex cases, or (2) engage in “creative coding” practices to shift patients into higher-paying DRG categories (7). The absence of significant insurance-type effects on surgical treatment selection (*p* = 0.79) in our multivariate model contrasts with earlier Chinese studies from the pre-DRG era, which documented substantial insurance-based disparities in access to surgery (21).

Our logistic regression analysis revealed that gender, age, and length of hospital stay were significant predictors of surgical intervention, with length of stay demonstrating the strongest association (adjusted OR = 106.729 for >16 days vs. <5 days). The pronounced gender disparity, with female patients exhibiting nearly twice the odds of undergoing surgery (adjusted OR = 1.989, 95%CI:1.624–2.436), warrants careful interpretation. This finding contradicts the traditional assumption that women are less likely to receive aggressive lung cancer treatment and aligns with recent evidence suggesting a paradigm shift in gender-based treatment disparities. Yang et al. (22) reported that women with non-small cell lung cancer (NSCLC) demonstrated better responses to surgical resection, particularly in adenocarcinoma subtypes, which may influence clinical decision-making toward more aggressive surgical management in female patients. Additionally, women tend to be diagnosed at earlier stages due to higher healthcare-seeking behaviors and better adherence to screening recommendations, making them more suitable.

surgical candidates (23). However, the observed association may also reflect residual confounding by unmeasured tumor characteristics (stage, histology, molecular markers) that were not available in our dataset-a critical limitation that requires acknowledgment.

The inverse relationship between age and surgical intervention, with patients aged >80 years showing 65% lower odds (adjusted OR = 0.353, 95%CI:0.161–0.774) compared to those ≤40 years, reflects well-established surgical risk stratification practices. Advanced age is associated with increased perioperative mortality, reduced physiological reserve, and higher prevalence of comorbidities such as cardiovascular disease and chronic obstructive pulmonary disease, which contraindicate major thoracic surgery (24). Recent evidence from China-specific cohorts corroborates this trend: Zheng et al. (25) demonstrated that among octogenarians with early-stage NSCLC, only 23% received surgical treatment compared to 68% of patients aged <70 years, largely due to concerns about functional outcomes and competing mortality risks.

Most strikingly, length of hospital stay emerged as the dominant predictor of surgical treatment, with patients hospitalized >16 days showing 107-fold higher odds of undergoing surgery. This finding requires nuanced interpretation beyond simple causality. While longer preoperative stays may reflect comprehensive staging workups, optimization of comorbidities, and neoadjuvant therapy administration-all prerequisites for complex surgical procedures (26), the association likely also captures postoperative recovery time. Surgical patients require extended stays for chest tube management, pain control, respiratory physiotherapy, and monitoring for complications such as air leaks, pneumonia, and atrial fibrillation (27). In contrast, non-surgical patients receiving palliative chemotherapy or supportive care typically have shorter admission durations (median 3–5 days in our cohort). Therefore, the observed association reflects both a marker of treatment complexity and a consequence of surgical intervention itself. The bidirectional relationship between hospitalization length and surgical treatment underscores the limitations of cross-sectional study designs in establishing temporal causality.

In conclusion, this study outlines key differences in medical expenses and influential factors impacting the treatment choices of lung cancer patients. Understanding these dynamics is essential for optimizing healthcare resource allocation, guiding policy decisions, and ultimately improving patient care in the context of lung cancer treatment.

The single-center retrospective design limits the generalizability of findings to other healthcare settings or regions in China and is susceptible to selection and information bias. The single-center retrospective design limits the generalizability of findings to other healthcare settings or regions in China and is susceptible to selection and information bias. The analysis lacks detailed clinical information such as cancer staging, histological subtypes, tumor size, and comorbidities, which are critical determinants of treatment selection and costs. Additionally, the study focused solely on hospitalization costs during a single calendar year (2023) without assessing post-discharge expenses, long-term survival outcomes, or quality of life measures, which may not capture temporal trends or seasonal variations in care patterns. The comparison between surgical and non-surgical groups did not include propensity score matching for baseline characteristics, and several potential unmeasured confounders such as patient preferences, physician decision-making patterns, hospital capacity, and reimbursement policies might influence treatment choices but were not accounted for. Furthermore, the study examined only direct medical costs without considering indirect costs such as productivity loss and caregiver burden, and while insurance type was included as a variable, detailed analysis of coverage policies and reimbursement rates was not performed. Finally, the study did not differentiate between various surgical approaches such as open thoracotomy versus video-assisted thoracoscopic surgery, which may have different cost implications.

## Data Availability

The datasets presented in this study can be found in online repositories. The names of the repository/repositories and accession number(s) can be found in the article/supplementary material.
